# LONG-TERM ASSOCIATION OF AN INTENSIVE LIFESTYLE INTERVENTION ON GLOBAL COGNITIVE FUNCTION

**DOI:** 10.1093/geroni/igad104.1699

**Published:** 2023-12-21

**Authors:** Mark Espeland, Kathleen Hayden, Stephen Rapp, Bonnie Sachs, Debbie Pleasants, Debbie Booth, Rena Wing, Rebecca Neiberg

**Affiliations:** Wake Forest University School of Medicine, Winston-Salem, North Carolina, United States; Wake Forest University School of Medicine, Winston-Salem, North Carolina, United States; Wake Forest University School of Medicine, Winston-Salem, North Carolina, United States; Wake Forest University School of Medicine, Winston-Salem, North Carolina, United States; Wake Forest University School of Medicine, Winston-Salem, North Carolina, United States; Wake Forest University School of Medicine, Winston-Salem, North Carolina, United States; Miriam Hospital, Providence, Rhode Island, United States; Wake Forest University School of Medicine, Winston-Salem, North Carolina, United States

## Abstract

The long-term effects of mid-life changes in lifestyle on later life cognitive function have not been rigorously assessed among individuals with type 2 diabetes. The Look AHEAD Aging study examined differences in standardized composite cognitive function scores in 1367 individuals, currently aged 64 to 95 years, who had enrolled an average of 20 years previously in a randomized controlled trial to compare a 10-year multidomain intensive lifestyle intervention (ILI) to a condition of diabetes support and education (DSE). The composite averaged standardized scores from the telephone administration of tests chosen to capture the individual cognitive domains of memory, attention/processing speed, executive function, and global cognitive function. At enrollment into the Look AHEAD Aging study there was no overall difference between intervention groups in cognitive test scores: mean [SE]: -0.01 [0.03] ILI versus 0.00 [0.04] DSE (p=0.76). However, among participants with overweight at Look AHEAD enrollment, those assigned to ILI had mean [95%CI] of 0.33 [0.08, 0.57] better global cognitive function scores than those assigned to DSE. For those initially with obesity, there was no relative intervention benefit: mean [95%CI] -0.08 [-0.18, 0.03] (interaction p=0.003). Benefits for those with overweight appeared to be strongest for tests of memory. ILI should be recommended to individuals with type 2 diabetes and overweight to provide long-term resilience against cognitive aging. The cognitive benefits we have reported earlier for individuals with overweight during intervention delivery endure for a decade following the cessation of the intervention.

